# Correlation between Cyclin Dependent Kinases and Artemisinin-Induced Dormancy in *Plasmodium falciparum In Vitro*

**DOI:** 10.1371/journal.pone.0157906

**Published:** 2016-06-21

**Authors:** Karen-Ann Gray, Karryn J. Gresty, Nanhua Chen, Veronica Zhang, Clare E. Gutteridge, Christopher L. Peatey, Marina Chavchich, Norman C. Waters, Qin Cheng

**Affiliations:** 1 Drug Resistance and Diagnostics, Australian Army Malaria Institute, Brisbane, Australia; 2 Clinical Tropical Medicine, QIMR Berghofer Medical Research Institute, Brisbane, Australia; 3 School of Biochemistry, University of Queensland, Brisbane, Australia; 4 US Naval Academy, Annapolis, Maryland, United States of America; 5 Walter Reed Army Institute of Research, Silver Spring, Maryland, United States of America; Université Pierre et Marie Curie, FRANCE

## Abstract

**Background:**

Artemisinin-induced dormancy provides a plausible explanation for recrudescence following artemisinin monotherapy. This phenomenon shares similarities with cell cycle arrest where cyclin dependent kinases (CDKs) and cyclins play an important role.

**Methods:**

Transcription profiles of *Plasmodium falciparum* CDKs and cyclins before and after dihydroartemisinin (DHA) treatment in three parasite lines, and the effect of CDK inhibitors on parasite recovery from DHA-induced dormancy were investigated.

**Results:**

After DHA treatment, parasites enter a dormancy phase followed by a recovery phase. During the dormancy phase parasites up-regulate *pfcrk1*, *pfcrk4*, *pfcyc2* and *pfcyc4*, and down-regulate *pfmrk*, *pfpk5*, *pfpk6*, *pfcrk3*, *pfcyc1* and *pfcyc3*. When entering the recovery phase parasites immediately up-regulate all CDK and cyclin genes. Three CDK inhibitors, olomoucine, WR636638 and roscovitine, produced distinct effects on different phases of DHA-induced dormancy, blocking parasites recovery.

**Conclusions:**

The up-regulation of PfCRK1 and PfCRK4, and down regulation of other CDKs and cyclins correlate with parasite survival in the dormant state. Changes in CDK expression are likely to negatively regulate parasite progression from G_1_ to S phase. These findings provide new insights into the mechanism of artemisinin-induced dormancy and cell cycle regulation of *P*. *falciparum*, opening new opportunities for preventing recrudescence following artemisinin treatment.

## Introduction

Although artemisinin-derivatives (ART) such as artesunate and dihydroartemisinin (DHA) exert a rapid action against malaria parasites, ART monotherapy is associated with frequent recrudescence [1]. An ART-induced dormancy phenomenon provides a plausible explanation for these ART monotherapy failures [2,3,4,5,6]. The dormancy phenomenon describes a subset of ART-treated ring-stage parasites entering a state of growth arrest that can last for a number of weeks before resuming growth when drug pressure decreases [2,3,4,6]. This subset of dormant parasites maintains metabolic activities in the apicoplast and mitochondria despite a general down-regulation in transcription of genes encoding key enzymes in various metabolic pathways [7,8]. This phenomenon is comparable to cell cycle arrest where cell growth stalls in response to unfavourable conditions, such as drug pressure or nutrient deprivation [9].

In mammalian cells, cell cycle progression and arrest are regulated by cyclin-dependent protein kinases (CDKs) [10]. A fully activated CDK requires association with a cyclin subunit and site-specific phosphorylation at a conserved threonine [11]. CDKs are inactivated by cyclin degradation, inhibitory phosphorylation within the ATP-binding domain, or binding of small inhibitory proteins. Timely activation and deactivation of the CDKs at each stage ensure cell cycle progression in a sequential manner. In mammalian cells, several CDKs and associated cyclins maintain the specific cell cycle phase. In *Saccharomyces cerevisiae*, however, a single CDK, CDC28 regulates multiple phases in association with different cyclin subunits [12].

While cell cycle phases and the associated regulatory mechanisms are well understood for mammalian and yeast cells, they are poorly defined for *Plasmodium* parasites. Development of ring-stage parasites into trophozoites signifies the G1 phase. DNA synthesis (S phase) begins as parasites develop through mature trophozoites into schizonts. Following DNA synthesis, mitosis (M phase) occurs resulting in nuclear division. Interestingly, the parasite appears to alternate between S and M phases through an endomitotic process resulting in numerous nuclei that subsequently become individual merozoites [13,14]. Unique features of the plasmodial cell cycle include an asynchronous cell cycle and an intact nuclear membrane during mitosis. Despite these unique features of the plasmodial cell cycle, many components of the eukaryotic cell cycle machinery have homologs in malaria parasites [15].

Several homologs of CDKs and cyclins are present in *P*. *falciparum* [16]. Amongst those are PfMRK and PfPK5, orthologues of human CDK7 and CDK1, respectively. Both PfMRK and PfPK5 are nuclear proteins that co-localize with replicating DNA [17,18] and play a role in the G_1_ and S phase of the cell cycle. Expression studies of various plasmodial CDKs and cyclins suggest that a PfMRK-PfCYC1 complex assembles during early ring-stage development prior to the initiation of DNA synthesis [19,20,21,22]. A correlation between inhibition of DNA replication and a decrease in PfPK5 activity suggests that kinase activity of PfPK5 is involved in initiation of DNA replication [18]. PfPK6, located in both the nucleus and the cytoplasm, is transcribed and active in late G_1_, S and M phases. PfPK6 appears to be a hybrid resembling both a CDK and MAPK, with significant kinase activity observed without a cyclin [23]. Other CDK-related kinases identified in *Plasmodium* are PfCRK1, PfCRK3 and PfCRK4. PfCRK1 is closely related to p58^*GTA*^-related kinase and is expressed in non-dividing gametocytes and asexual stages [24]. The homolog of PfCRK1 in *P*. *berghei* is essential for parasite growth [25]. PfCRK3 has been demonstrated to interact with a histone deacetylase and is essential for parasite proliferation [26]. Based on transcription data, PfCRK1 may function during the S phase (late trophozoite), whereas PfCRK3 and PfCRK4 functions during the G_1_ phase (early rings), and late schizogony (mitosis), respectively, in *P*. *falciparum* [27].

Four cyclin encoding genes, *pfcyc1-4*, have been identified in *P*. *falciparum* [19,22]. Unlike mammalian cyclins, plasmodial cyclins promiscuously bind and activate various CDKs: PfCYC1 and PfCYC3 bind and activate PfPK5 [19,22] while PfCYC1 binds and activates PfMRK. Functions of PfCYC2 and PfCYC4 are unclear.

Several mammalian CDK inhibitors have been used to characterize plasmodial CDKs. Roscovitine, an inhibitor of mammalian CDK1, CDK2 and CDK5, inhibits activities of PfPK5 [28] and PfPK6 [23], while olomoucine, an inhibitor of CDK1 and ERK1, inhibits kinase activity of recombinant PfCRK1 [29]. Although both roscovitine and olomoucine inhibit activities of recombinant PfPK6, roscovitine has six times greater potency against PfPK6 than olomoucine [23]. Both olomoucine and roscovitine fail to inhibit PfMRK [30]. Conversely, chalcones have been shown to effectively inhibit PfMRK [31,32], not PfPK5 [33].

Of note, ART derivatives also possess anticancer properties [34] and have been reported to induce G1 phase arrest in several cancer cell lines including choriocarcinoma [35], hepatoma [36] and prostate cancer [37]. For instance, artesunate produces a stringent G_1_ arrest of prostate cancer growth which was associated with down-regulation of CDK4 and CDK2 [37].

We hypothesize that ART-induced dormancy functions through a cell cycle arrest mechanism in *Plasmodium* and that cell cycle machinery including CDKs and cyclins, play an important role in this process. To test this hypothesis we investigated the transcription profiles of plasmodial CDKs and cyclins during DHA-induced dormancy. The activities of CDKs and cyclins during DHA-induced dormancy were further investigated using CDK inhibitors. The results show that different CDKs are involved in parasites entering and exiting DHA-induced dormancy. The likely function of these CDKs during dormancy is blocking transition of parasites from G_1_ to S phase. These findings provide new insights into parasite cell cycle regulation in ART-induced dormancy.

## Materials and Methods

### In vitro cultivation and synchronization of *P*. *falciparum* lines

W2 (Indochina), D6 (Serra-Leone) and S55 (Solomon Islands) *P*. *falciparum* lines were maintained in vitro at 3% haematocrit using RPMI1640 medium supplemented with 10% human plasma [38]. Parasites were synchronized using D-sorbitol [39] at ring-stage and MACs column (MACS Miltenyi Biotec) at mature stages [2]. These procedures were repeated during two consecutive parasite life cycles.

### Harvest of untreated parasites for transcription analyses

Each synchronized parasite line was split into six 10 ml flasks. Untreated ring-stage parasites at 2% parasitemia were cultured under standard conditions. One flask was harvested at 12-hourly intervals (0, 12, 24, 36, 48 and 60 h) of which 1 ml was used for RNA preparations. Time 0 h relates to the time when treatment started in treated parasites.

### Harvest of DHA treated parasites for transcription analysis

120 ml of synchronized ring-stage parasites at 2% parasitemia was treated with DHA (Haphagen, Hanoi, Vietnam) at 200 ng/mL (1 mM stock was prepared in 100% methanol) for 6 h (starting at time 0 h) then washed using complete medium and resuspended in 120 ml media. On days 1–3 post-treatment, the treated parasites were passed though MACs columns as described [2] and resuspended to the starting volume, then, a 10 ml culture was harvested each day. The remaining culture was split into 9 flasks (10 ml each) after day 3. One flask was harvested every day between days 4 and 12 from each parasite line of which 1 ml was used for RNA preparation.

### Determination of parasite density in the culture for transcription analysis

100 μL of culture was removed at each time point when untreated and treated parasites were harvested, and used to estimate parasite density by staining with SYBR Green (SG) followed by FACS analysis [8] or by microscopy analysis of Giemsa stained slides.

### Isolation of total RNA

The untreated and treated 1 mL parasite fractions were lysed using saponin (0.15%) for 5 min on ice and the pellet washed 2–3 times with PBS, resuspended in 350 μL of RA1 buffer plus 3.5 μL β-mercaptoethanol and stored at -70°C. Total RNA was isolated using the NucleoSpin RNA II kit (Macherey-Nagel). The isolation step was repeated once. Total RNA was eluted with 60 μL of RNase free water and was treated with DNase (Promega) and RNasin (Promega) following manufacturer’s instructions. To ensure the total RNA does not contain DNA a house-keeping gene, the seryl-tRNA synthetase gene (*pfsars*), was amplified with and without reverse transcriptase.

### cDNA synthesis and quantitative real-time (qRT)-PCR

cDNA was synthesised using Superscript III reverse transcriptase (Invitrogen), 0.5 μL random primer mix (500 ng/μL, Promega), 4 μL of dNTP mix (2.5 mM each dNTP) and 5 μL of total RNA following manufacturer’s instructions. Gene specific primers designed to amplify 10 genes encoding plasmodial CDKs and cyclins were used in qRT-PCR to quantify transcripts of these genes after their amplification efficiency were determined to be similar (Table 1). *Pfsars* was amplified from each samples as a positive control for qRT-PCR. qRT-PCR was performed in triplicate using FastStart Essential DNA green master mix (Roche), 1 μL of cDNA and 1 μL of each primer (5 μM) in a 25 μL reaction under conditions of 95°C for 10 min followed by 45 cycles 95°C for 10 sec, 60°C for 20 sec and 72°C for 10 sec in a LightCycler96 (Roche). A standard curve, using serially diluted 3D7 genomic DNA (10-fold dilution from 4 to 0.004 ng/μL) was included in each qRT-PCR.

**Table 1:** *P*. *falciparum* gene names (abbreviation), ID numbers and primers used for amplification of CDKs and cyclins. The seryl-tRNA synthetase gene was used as a positive control.

Gene name (abbreviation) Gene ID	Sequences 5’ to 3’
Pf seryl-tRNA synthetase (Pfsars) Pf3D7_0717700	Pfsars-f AAGTAGCAGGTCACGTG
	Pfsars-r CGGCACATTCTTCCATA
Pf MO15-related protein kinase (Pfmrk) Pf3D7_1014400	Pfmrk-f TGTGGAGTTTTGGTTGTATTTTT
	Pfmrk-r TTCTGGCCAATTATTTTCGTTAG
Pf Protein Kinase 5 (pfpk5) Pf3D7_1356900	Pfpk5-f TCGAGCACCAGATGTTTTAATGGGA
	Pfpk5-r TGGTCTGCTTCAGATACCCCTGGA
Pf Protein Kinase 6 (pfpk6) Pf3D7_1337100	Pfpk6-f AGTGACCACATGACACCTACTGT
	Pfpk6-r CCATGAATAAACAACCCAAACTCCA
Pf cdc2 related protein kinase 1 (pfcrk1) Pf3D7_0417800	Pfcrk1-f CGTTCACACTTCCCAAATATAGCCA
	Pfcrk1-r AGGATGGTTTAATGCTTCCTGTGCT
Pf cdc2 related protein kinase 3 (pfcrk3) Pf3D7_0415300	Pfcrk3-f ACGGAAACCACAAATATGGAGCAGA
	Pfcrk3-r TCCCTATCACTGTAATCCGTGACCG
Pf cdc2 related protein kinase 4 (pfcrk4) Pf3D7_0317200	Pfcrk4-f AAAGAAGAGCATGTTGAAAAGGGCA
	Pfcrk4-r TTCGTCATGCCACTTTTATTGGTGT
Pf cyclin 1 (pfcyc1) Pf3D7_1463700	Pfcyc1-f CGCAAATGCGGATATGTTTATCT
	Pfcyc1-r ACCGATTTGTCTGGTCGTATTT
Pf cyclin 2 (pfcyc2) Pf3D7_1227500	Pfcyc2-f AAGTCGGTGGACGAGACAGC
	Pfcyc2-r TGGGGGACAAATCATCTACTTCGT
Pf cyclin 3 (pfcyc3) Pf3D7_0518400	Pfcyc3-f AGGGAGAAGTACCAAGAACCGA
	Pfcyc3-r TCAGGAACTTTAGAAGCATGAAAAC
Pf cyclin 4 (pfcyc4) Pf3D7_1304700	Pfcyc4-f ACATCCTCATTCATTTCTTTTACCA
	Pfcyc4-r TCCCCAAGACATTTGTGCTA

### Analysis of qRT-PCR data

The average quantification cycle (Cq) value was calculated for each gene examined at each time point. These values were converted to RNA concentrations based on the standard curve and normalised against the parasite density of the sample determined by SG as previously published [7]. Transcriptions of house-keeping genes could not be used as normalisers because their transcriptions were down regulated during the DHA induced dormancy [7]. The transcription level of each gene in untreated rings (day 0) was set as 1 and the transcription level of each gene at the remaining time points (both untreated and treated) expressed as fold changes relative to day 0 baseline [7].

### Effect of kinase inhibitors on DHA-induced dormancy

Roscovitine (Sigma-Aldrich), olomoucine (Sigma-Aldrich) and WR636638 (Walter Reed Army Institute of Research) were used separately at their respective IC_50_ values to treat W2 parasites for 48 h. IC_50_ of inhibitors for W2 were determined in a standard 48 h in vitro susceptibility test using SG dye: roscovitine (7.15 μg/mL), olomoucine (174.74 μg/mL) and WR636639 (16.01 μg/mL). Two independent experiments were conducted in triplicate of 1 ml each at a starting parasitemia of 2%. Each inhibitor was added at different times during the dormancy experiment: a) together with DHA on day 0, so the parasites were treated with both DHA (200 ng/mL) and the inhibitor for 6 h, then washed off, following by the addition of the inhibitor for a further 42 h; b) post-DHA treatment on day 2, 4 and 6, respectively, as described previously [7]. Parasites treated with either DHA or the CDK inhibitor alone were used as controls for dormancy and growth inhibition effects. Parasite density of treated parasites was determined by microscopy.

### Human ethics

Human erythrocytes and plasma used for in vitro cultivation of *P*. *falciparum* was provided *by* Australian Red Cross Blood Service in Brisbane. The use of human blood for in vitro culture of malaria parasites was approved by the Australian Defence Joint Health Command Low Risk Ethic Panel (LREP 15–014).

## Results

### Development of parasite stages in untreated controls

**Fig 1:**
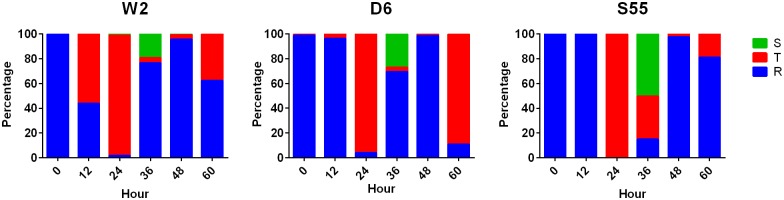
Percentage of life cycle stages of *P*. *falciparum* lines of W2, D6 and S55 at different time points (0, 12, 24, 36, 48 and 60 h). S, T and R represents schizont, trophozoite and ring-stages, respectively.

At 0 h between 99 and 100% of W2, D6 and S55 parasites were at ring-stage. After 24 h, 96–100% of parasites were trophozoites. At 36 h, between 20 and 50% of parasites were schizonts. At 48 h, 95 to 98% of parasites were new rings (Fig 1).

### Transcription profiles of CDKs and cyclins in untreated parasites

**Fig 2:**
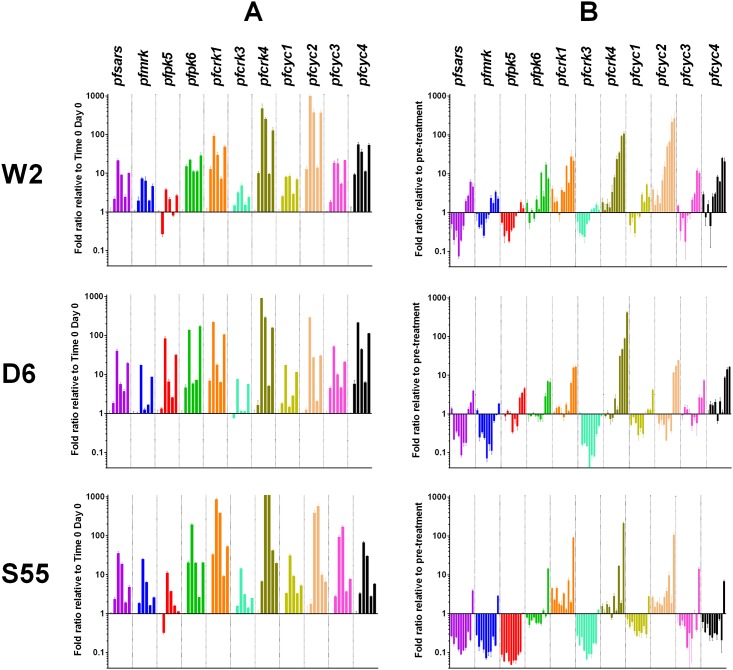
Transcription levels of CDK and cyclins (shown in different colours) in untreated and DHA treated *P*. *falciparum* W2, D6 and S55 parasites. Panel A: untreated parasites. Transcription levels of each gene at 12, 24, 36, 48 and 60 h are grouped from left to right and are expressed as fold ratio relative to time 0. Panel B: DHA treated parasites. Transcription levels of each gene between day 1 and day 10 are grouped from left to right and are expressed as fold ratio relative to pre-treatment (time 0 h of untreated parasites on Day 0). Error bars represent standard error.

Transcription of all 10 genes increased between 0 h and 24 h except for *pfpk5* in W2 and S55 at 12 h. Transcription of 10, 9 and 8 genes in W2, D6 and S55, respectively, peaked at 24 h when parasites were at the trophozoite stage (Figs 1 and 2A). Transcription levels decreased from 36 h to 60 h (schizont to the next trophozoite stage, Figs 1 and 2A). Overall, transcription levels of *pfpk6*, *pfcrk1*, *pfcrk4*, *pfcyc2*, *pfcyc3 and pfcyc4* were higher than *pfsars*, while the transcription levels of *pfmrk*, *pfpk5*, *pfcrk3 and pfcyc1* were lower than *pfsars*, although the rank order of the transcription level of each gene varied slightly between the three parasite lines (Fig 2A).

### Dynamics of parasitemia post-DHA treatment

**Fig 3:**
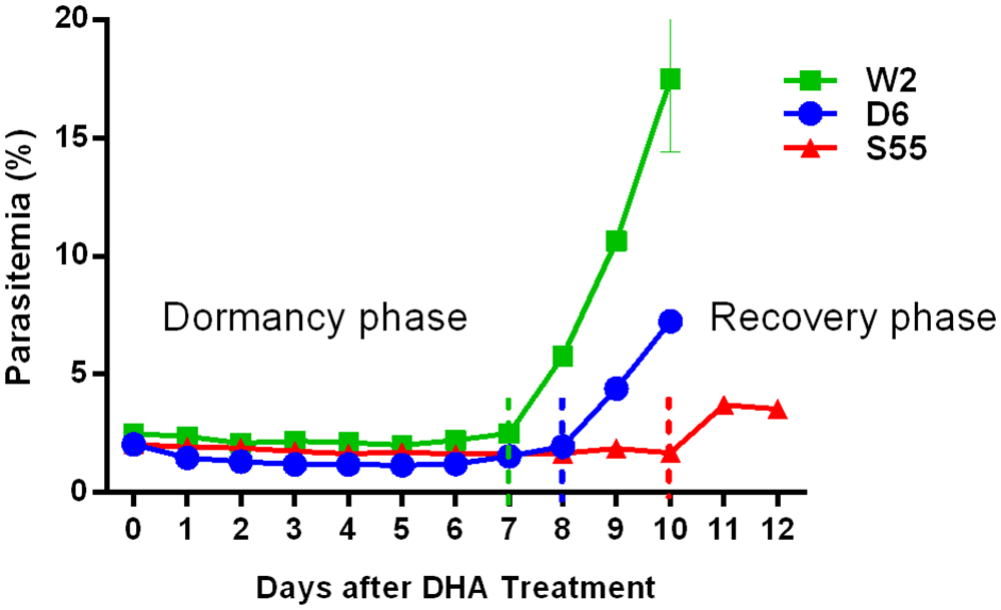
Dynamics of parasite density following DHA treatment of *P*. *falciparum* lines of W2, D6 and S55. Parasitemia are determined by SYBR Green staining and FACS analysis. Dotted lines indicate boundaries between dormancy and recovery phase in each parasite line.

Following treatment, parasite density measured by SG was at pre-treatment levels with some parasites remaining dormant for 7–10 days as previously demonstrated [2,8]. W2, D6 and S55 parasite recovery, signified by an increase in parasite densities, began on days 7, 8 and 10, respectively (Fig 3). Therefore, W2, D6 and S55 parasites were classified in the dormancy phase (dormancy) during days 1–6, 1–7 and 1–9, respectively, and in the recovery phase (recovery) after days 7, 8 and 10, respectively (Fig 3).

### Transcription profiles of CDK and cyclin transcripts during DHA-induced dormancy

Following DHA treatment, transcription of *pfsars* (a house keeping gene) was markedly down-regulated in all three parasite lines during dormancy, as previously shown [7], only returning to pre-treatment level and above at the beginning of recovery. Similarly, transcription of *pfmrk*, *pfcrk3* and *pfcyc1* was down-regulated in all three parasite lines during dormancy but increased during recovery (Fig 2B). Transcription of *pfpk5*, *pfpk6* and *pfcyc3* was also down-regulated during dormancy in W2 and S55, but remained at pre-treatment levels for the first 4 days of dormancy in D6 (Fig 2B).

In contrast, transcription levels of *pfcrk1* and *pfcrk4* were at pre-treatment levels or up-regulated in all three parasite lines during dormancy (Fig 2B). Greater than 5-fold increases in transcription for both genes were observed during recovery. Transcription of *pfcyc2* was also up-regulated in W2 and S55, but was down-regulated in D6, whereas transcription of *pfcyc4* was at pre-treatment level or up-regulated in W2 and D6, but was down-regulated in S55 during dormancy (Fig 2B). Interestingly, in all parasite lines, at least one CDK and a cyclin were up-regulated during dormancy. Whether this represents an active CDK-complex regulating some cellular function during dormancy warrants further investigation.

### Effect of kinase inhibitors on the dynamics of recovery from DHA-induced dormancy

W2 was chosen for this experiment because of its propensity for earlier recovery after DHA treatment (Fig 3). Untreated parasites, parasites treated with DHA alone and CDK inhibitor alone were used as controls for the experiment.

**Fig 4:**
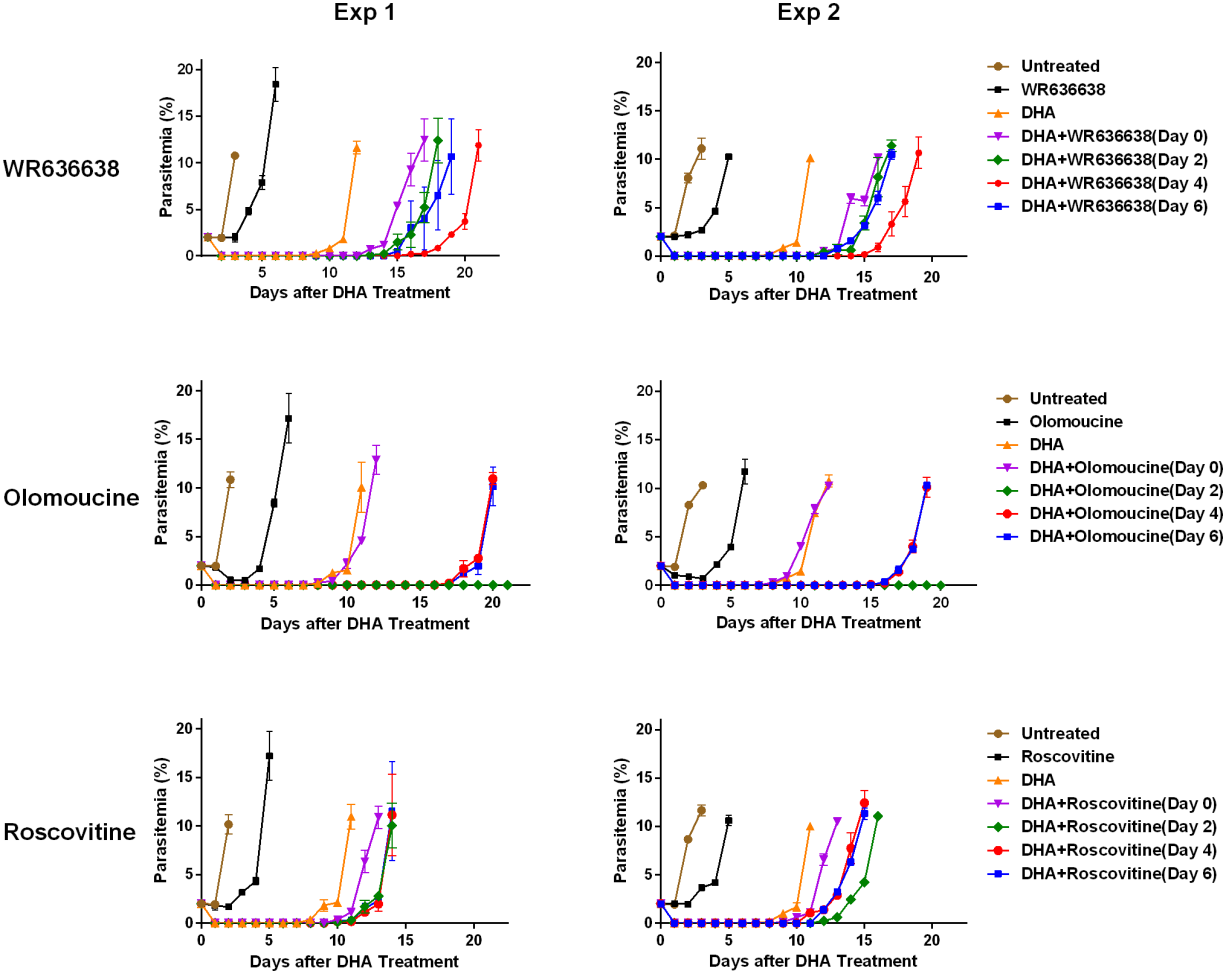
Effect of the CDK inhibitors (WR636638, olomoucineon and roscovitine) on untreated and DHA-treated W2 parasites. Parasites were treated with DHA alone for 6 h, or one of the CDK inhibitors alone for 48 h (at Day 0), or DHA (for 6 h) plus one of the CDK inhibitors (for 48 h). For DHA plus CDK inhibitors, one of the CDK inhibitors was added either together with DHA (Day 0) or after DHA treatment (Days 2, 4 and 6) for 48 h. Two panels (Exp 1 and Exp 2) represent results of 2 independent experiments, each in triplicate. Parasitemia (percentages of parasitized erythrocytes by morphologically normal parasites) was determined by microscopy.

Among the control groups, the untreated W2 parasites reached 10% parasitemia on day 2. Following treatment with DHA alone for 6 h, parasites arrested their growth for 8 days and then recovered with rising parasite density on day 9 (Fig 4). Treatment with each of the three CDK inhibitors alone at their IC_50_ for 48 h delayed parasite growth by 3 days, with parasite density reaching 10% between days 5 and 6 (Fig 4). Treatment of parasites using these inhibitors alone at the concentrations used did not cause parasites to enter dormancy.

The three CDK inhibitors, either added together with DHA (day 0) or during dormancy (days 2, 4, or 6), demonstrated different effects on the dynamics of DHA-induced dormancy. WR636638, when added together with DHA (day 0) or on days 2, 4 or 6 after DHA treatment, prolonged the duration of dormancy. Its addition on day 4 post-DHA treatment for 48 h produced the strongest blocking effect delaying recovery to 10% parasitemia by 8–10 days (Fig 4). Interestingly, of the three inhibitors tested, WR636638 had the most pronounced effect when combined with DHA treatment.

Olomoucine showed a strong effect on parasite recovery when added on days 2, 4 or 6 during dormancy for 48 h, but did not show an effect when added together with DHA. When added on day 2 post-DHA treatment, olomoucine prevented parasite recovery until the end of the experiment (day 21). When added on days 4 and 6 after DHA treatment, olomoucine delayed parasite recovery to 10% by 7–9 days (Fig 4).

Roscovitine showed the least effect on dormancy recovery. Its addition resulted in a moderate delay of 3–4 days for parasite recovery to 10% as compared to DHA treatment alone (Fig 4).

## Discussion

Although the mechanism of ART-induced dormancy is not clear, its similarity to cell cycle arrest seen in mammalian cells suggests that cell cycle regulators such as CDKs and cyclins would play important roles in parasites entering and exiting dormancy. The focus of this study was to investigate the possible role CDKs and cyclins play at various phases of DHA-induced dormancy.

The first question addressed was what CDKs and cyclins were expressed during DHA-induced dormancy and recovery in ART sensitive parasites. This was done by examining the transcription profiles of 10 genes, encoding six CDKs and four cyclins, before and after DHA treatment when DHA-treated parasites were at different stages of the dormancy and recovery phase (days 0–12). Therefore, this study differs to published transcriptional profiling studies where parasites were either untreated [40,41] or treated with artesunate [42] or DHA [43] for 1–3 hours and were immediately analysed for transcription before dormant parasites were seen. Immediately after 1–3 hr artesunate treatment up- or down regulation was observed for 4 cell cycle related genes, but no significant changes were observed for the set of CDKs and cyclins examined in the present study [42]. However, after 3 hr DHA treatment only a significant up-regulation (<2 fold) in *pfcrk3* transcription was seen [43]. These changes may reflect parasites immediate response to ART treatment.

In the present study, transcription of all CDKs and cyclins were detected in untreated parasites with levels peaking at trophozoite stage. After DHA treatment, parasites enter dormancy for 7–10 days, followed by recovery. During the dormancy phase, transcription of four CDKs (*pfmrk*, *pfpk5*, *pfpk6*, *pfcrk3)* and two cyclins (*pfcyc1* and *pfcyc3*) were down-regulated in all three parasite lines, but then increased during recovery. As PfCYC1 interacts with PfMRK and PfPK5 [19,20,21,22] the parallel down-regulation of *pfcyc1*, *pfmrk* and *pfpk5* is expected. In contrast, transcription of *pfcrk1* and *pfcrk4* was up-regulated throughout dormancy and recovery in all three parasite lines. The increase in transcript levels of these genes is likely to be a result of an increase in their transcription, rather than accumulation of earlier synthesised transcripts, as their half-lives have been shown to be similar to the other CDKs in untreated parasites [44] although their stability may be altered by DHA treatment and we could not rule this out. Similarly, *pfcyc2* and *pfcyc4* also remained at pre-treatment levels or up-regulated throughout dormancy in two of the three parasite lines. It is possible that PfCYC2 and PfCYC4 are the cognate cyclins responsible for activating PfCRK1 and PfCRK4. Previously, PfCYC4 has been categorized as a member of cyclin L family that would bind PfCRK1 [45]. Future studies are needed to confirm their association. The presence of a cyclin-CDK complex may either be responsible for jump-starting the cell cycle in recovering parasites or for suppressing cell cycle progression.

Among the four down-regulated CDKs, PfMRK, PfPK5 and PfPK6 have been identified as homologs of human CDK7, CDK1 and CDK2 respectively, and their activities have been associated with initiating DNA synthesis [17,18,19,20,21,22,23,46]. PfCRK3 has been associated with parasite proliferation [26]. Hence, the down-regulation of these CDKs observed during dormancy would be expected to halt DNA synthesis, thus preventing parasite progression from G_1_ to S phase. The down regulation of PfMRK after DHA treatment is supported by an earlier study reporting a remarkable down regulation of CDK-activating kinase assembly factor after artesunate treatment [41], which has been shown to form a complex with, and to activate PfMRK [46]. In contrast, PfCRK1, a homolog of human p58^*GTA*^-related kinase, a negative regulator of cell growth, was observed to be consistently up-regulated during dormancy. Although the function of PfCRK4 in *P*. *falciparum* is unknown, it is tempting to hypothesize that it may also be a negative cell cycle regulator. The observed correlation between the up-regulation of *pfcrk1* and *pfcrk4*, the down-regulation of the other four CDKs and dormancy suggest that these changes may be required by parasites entering and surviving dormancy.

These CDK roles were further substantiated by the distinct effects of three CDK inhibitors on parasite recovery from DHA-induced dormancy. Of the three inhibitors, olomoucine produced the strongest effect on parasite recovery from dormancy when added after DHA treatment. It completely blocked parasite recovery when added on day 2 post-DHA treatment and delayed parasite recovery when added on days 4 or 6. However, olomoucine had no effect when added together with DHA. These observations suggest that the target(s) of olomoucine plays an essential role in the survival and recovery of dormant parasites, rather than in triggering dormancy. In contrast to olomoucine, roscovitine only had a moderate effect on the parasite recovery from dormancy.

The sharp contrast between the effects of these CDK inhibitors suggests that a unique target(s) of olomoucine, rather than those common to both olomoucine and roscovitine, is essential for survival of dormant parasites. Both olomoucine and roscovitine fail to inhibit PfMRK however olomoucine has been shown to inhibit PfCRK1 [29] and PfPK5 [18] and to a lesser extent PfPK6 [23,47], whereas roscovitine predominantly inhibits PfPK5 and PfPK6 [23,28]. Taken with the findings that transcription of *Pfcrk1* is up-regulated in dormant parasites, these data suggest an important regulatory role for PfCRK1 in dormant parasites. Further supporting this, a homolog of PfCRK1, p58GTA kinase, acts as a negative regulator of cell growth in mammalian cells, as its over-expression inhibits entry into the S phase [48]. *Pfcrk1* has been reported to be expressed predominantly in non-dividing gametocytes [24] and was implicated in cell cycle-arrest. *Pfcrk1* is also expressed in asexual blood stages [27,49]. Therefore, we hypothesize that PfCRK1 plays an important role in controlling and suppressing parasite entry into S phase and is essential for survival of dormant parasites.

WR636638, a chalcone compound, also significantly delayed parasite recovery from dormancy. It was the only compound that delayed parasite recovery in combination with DHA. Its addition on days 2, 4 or 6 post-DHA treatment also delayed recovery from dormancy with the strongest effect observed when it was added on day 4. As chalcones effectively inhibit PfMRK [31,32], not PfPK5 [33], the result suggests that PfMRK plays an important role in parasites entering and exiting from dormancy. Since PfMRK has been implicated in initiating DNA synthesis [17], its down-regulation and activity inhibition is likely to block transition of ring-stage parasites (G_1_ phase) into trophozoites (S phase) driving them into a dormant state. It is conceivable that further suppression of PfMRK by WR636638 later in dormancy (days 4 and 6) results in fewer parasites entering S phase or recovery. Distinct effects produced by the three CDK inhibitors on different phases of DHA-induced dormancy are consistent with cell cycle phase-specific CDK activities, suggesting that these modulation of CDK activity results in a delay in parasite recovery.

This study, however, has some limitations. Due to the lack of highly specific antibodies to each CDK, we could not directly demonstrate an increase in activities of PfCRK1 and PfCRK4 or a decrease in activities of other CDKs corresponding to their transcription levels. In addition, the three CDK inhibitors used in this study, olomoucine, rocovitine and WR636638, may have off-target effect against molecules other than CDKs due to their relaxed selectivity. Therefore, the exact roles played by CDKs in DHA-induced dormancy require confirmation in future studies.

Based on the role of CDKs in higher eukaryotes and in *Plasmodium* and results of this work, we hypothesize that following exposure to DHA, parasites increase transcription and expression of PfCRK1 and PfCRK4 to exert a negative control to stop parasite progression into the S phase. Parasites also down-regulate PfMRK and other CDKs to prevent the initiation of DNA replication. Through the effect of these regulators, parasites fail to make transition from G_1_ to S phase and arrest at the G_1_ phase. These findings provide new insights into the cell cycle regulation of *P*. *falciparum* and most importantly, the mechanism of DHA-induced dormancy. Furthermore, the transcription profiles of *pfcrk1* and *pfcrk4* could be used in conjunction with transcription profiles of several apicoplast and mitochondria genes reported earlier [7,8] as markers for dormant parasites. Additionally, our data may reveal new antimalarial drug targets and provide molecular markers for dormant parasites, thus opening new possibilities for preventing recrudescence following artemisinin treatment.
